# The Chemical Composition of Brazilian Green Propolis and Its Protective Effects on Mouse Aortic Endothelial Cells against Inflammatory Injury

**DOI:** 10.3390/molecules25204612

**Published:** 2020-10-10

**Authors:** Xiaolan Xu, Bo Yang, Danfeng Wang, Yuxuan Zhu, Xiaoqing Miao, Wenchao Yang

**Affiliations:** 1 College of Animal Science (College of Bee Science), Fujian Agriculture and Forestry University, Fuzhou 350002, China; xlxufz@fafu.edu.cn; 2 College of Food Science, Fujian Agriculture and Forestry University, Fuzhou 350002, China; 15225700066@163.com (B.Y.); z18350993003@126.com (Y.Z.); 3 Bee Product Processing and Application Research Center of the Ministry of Education, Fuzhou 350002, China; mxqsf88@126.com; 4 College of Plant Protection, Institute of Applied Ecology, Fujian Agriculture and Forestry University, Fuzhou 350002, China; 91136459@163.com

**Keywords:** Brazilian green propolis, chemical composition, mouse aortic endothelial cells, anti-inflammatory

## Abstract

Propolis has a very complex composition, with antibacterial, anti-inflammatory and other properties. To determine the composition of ethanol extracts of Brazilian green propolis (EEP-B) and their protective effect on mouse aortic endothelial cells (MAECs), the chemical composition of EEP-B was analysed by UPLC/Q-TOF-MS/MS, and the protective effect of EEP-B on the proliferation of lipopolysaccharide (LPS)-induced MAECs was determined by Cell Counting Kit-8 (CCK-8) assays. The protein levels of inflammatory cytokines tumour necrosis factor-α (TNF-α) and interleukin- 6 (IL-6) were measured by enzyme-linked immunosorbent assay (ELISA), and ICAM-1, VCAM-1 and MCP-1 expressions were analysed by western blotting. The results showed that a total of 24 compounds belonging to cinnamic acids and flavonoids, including 3,5-diisopentenyl-4-hydroxycinnamic acid (artepillin C), kaempferide, 3-isoprenyl p-coumaric acid, pinocembrin and 4′-methoxy pinobanksin, were identified in EEP-B. Among them, a new component, suggested to be 5-isoprenyl caffeic acid p-coumaric acid ester, was reported for the first time. The LPS-induced levels of TNF-α, IL-6, ICAM-1, VCAM-1 and MCP-1 were downregulated in response to 5, 10 and 20 μg/mL EEP-B. This study revealed that EEP-B could reduce LPS-induced inflammatory reactions, improve cell survival, and protect MAECs by regulating ICAM-1, VCAM-1 and MCP-1 expression. These findings could provide a theoretical basis for MAEC treatment using EEP-B.

## 1. Introduction

Propolis contains very complex chemical components, mainly flavonoids and polyphenols, which vary depending on the geographical location, plant species and season [1,2,3]. The main plant sources of propolis are *Populus* spp. (*Salicaceae*) [4], *Baccharis dracunculifolia* [5], *Dalbergia ecastaphyllum* [6], Clusia (*Clusiaceae*) [7] and *Azadirachta indica* [8], resulting in the propolis composition being particularly complex. Among these, *Baccharis dracunculifolia* is the plant source of Brazilian green propolis, which contains isopentenylated phenylpropanoids and their derivatives, caffeoylquinic acids and some terpenes [9,10,11]. In contrast to poplar-type propolis, Brazilian propolis is mainly composed of phenolic acids and has an especially high level of artepillin C (3,5-diisopentenyl-4-hydroxycinnamic acid) [12,13].

Propolis has been demonstrated to have many pharmacological properties, including antimicrobial, antioxidant, anti-tumour, anti-inflammatory and immunomodulatory activities [14,15,16,17]. In particular, propolis has an anti-inflammatory effect, which can prevent atherosclerosis, reduce blood lipids, affect angiogenesis, and play an important role in the prevention and treatment of cardiovascular diseases [18]. Previous studies have shown that propolis could inhibit inflammation by inhibiting inflammatory cytokines (TNF-α, IL-1β, and IL-6), the ERK1/MAPK pathway, NF-κB activation, and neutrophil adhesion and transmigration (ICAM-1, VCAM-1 and E-selectin expression) [19]. Propolis extract could also effectively inhibit the levels of total cholesterol (TC), triglyceride (TG), non-high-density lipoprotein cholesterol (non-HDL-C), and the production of IL-6 and IL-17 in ApoE−/−mice fed a high-fat diet to protect vascular endothelial cells [20]. Some active ingredients in propolis, such as artepillin C [21,22], neovestitol–vestitol [23] and chrysin [24,25], have been proven to have strong physiological activity. As the most important component in green propolis, artepillin C exerted robust antioxidant and anti-inflammatory activity to inhibit the levels of the cytokines TNF-α, IL-1*β* and NF-𝜅B in stimulated RAW264.7 macrophages [26].

The vascular endothelium is a single layer of cells lining the surface of the vascular lumen that can maintain the integrity of blood vessels and the normal structure and function of the blood vessel wall [27]. Endothelial damage can be caused by oxidative stress, inflammation, hypertension and other factors [28]. As a strong inflammatory promoter, lipopolysaccharide (LPS) can stimulate inflammation and the release of a large number of proinflammatory cytokines, such as TNF-α, IL-1β, IL-6 and IL-10 [29,30], which are involved in the formation of atherosclerotic lesions. Currently, protecting vascular endothelial cells and regulating the inflammatory response of vascular endothelial cells are important treatments for atherosclerosis.

Brazilian green propolis has a complex chemical composition and strong anti-inflammatory effects. In this study, to determine the composition of Brazilian green propolis and its protective effect on LPS-stimulated mouse aortic endothelial cells (MAECs), the components of Brazilian green propolis were analysed by UPLC-Q-TOF-MS, LPS was used to stimulate MAECS, and the levels of TNF-α, IL-6, ICAM-1, VCAM-1 and MCP-1 were determined after propolis treatment to explore the protective effect of propolis on MAECS injured by LPS, as well as the possible mechanism.

## 2. Results

### 2.1. Chemical Composition

**Figure 1:**
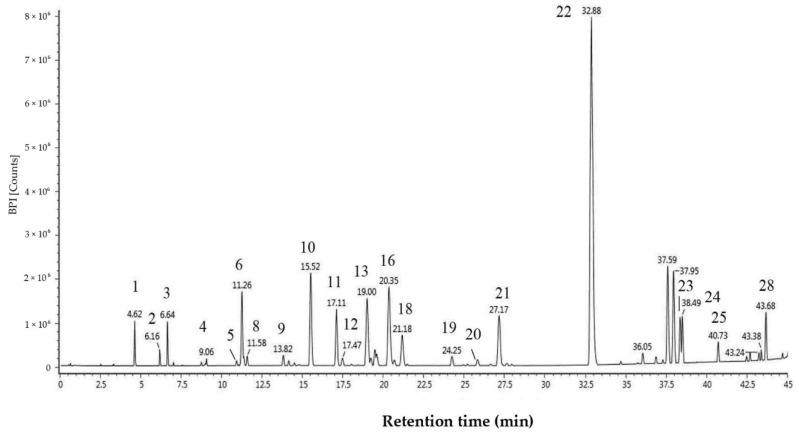
Chromatographic profile of 5-isoprenyl caffeic acid p-coumaric acid ester in Brazilian propolis.

The levels of total polyphenols and flavonoids in EEP-B were 218.67 mg/g and 227.20 mg/g, respectively. Chromatographic profiles and chemical compositions were determined by UPLC-Q-TOF-MS, and are shown in Figure 1 and Table 1. A total of 28 constituents was identified in EEP-B. These components mainly belong to cinnamic acids, of which artepillin C was the most abundant component (relative content 35.68%). In addition, there were four triterpenes detected, but their structures have not been identified.

**Figure 2:**
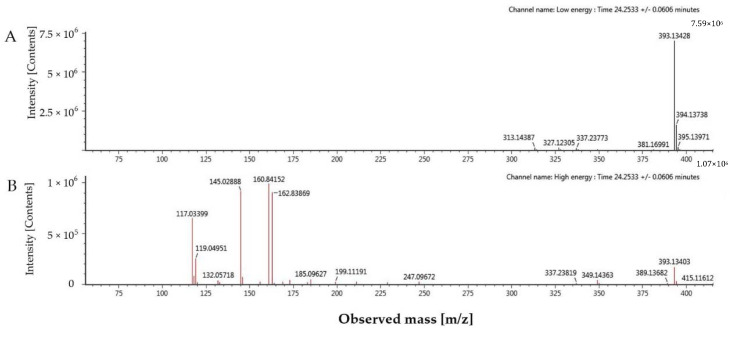
MS/MS spectra under low energy (**A**) and high energy (**B**) of 5-isoprenyl caffeic acid p-coumaric acid ester.

**Figure 3:**
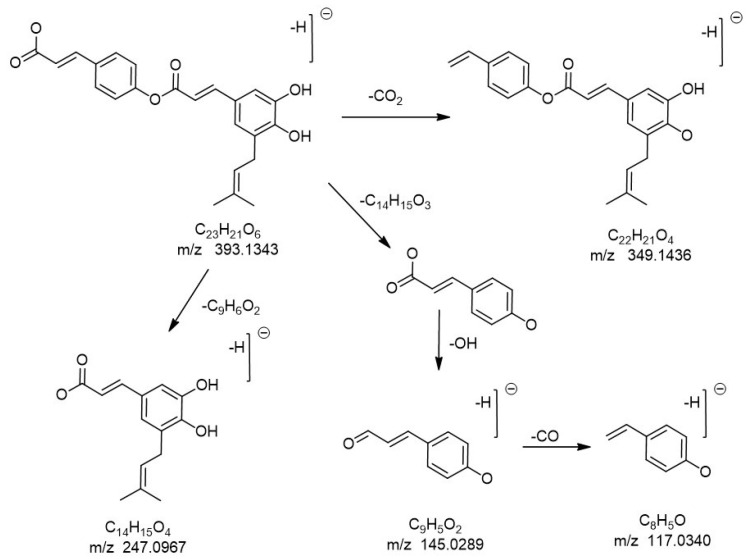
The fragmentation pathways of 5-isoprenyl caffeic acid p-coumaric acid ester.

In this study, a new compound (tR = 24.25 min) with a molecular formula of C_23_H_22_O_6_ was tentatively identified. The molecular ion at *m*/*z* 393.1343 [M–H]^–^ further fragmented to yield ions at *m*/*z* 349.1436 [M–H–CO_2_]^–^ and 247.0967 [M–H–C_9_H_6_O_2_]^–^. This compound also gave fragments at *m*/*z* 162.8387 [M–H–C_14_H_15_O_3_], 145.0289 [M–H–C_14_H_15_O_3_–OH]^–^ and 117.0340 [M–H–C_14_H_15_O_3_–OH–CO]^–^ by continuous fragmentation (Figure 2). As a result, its chemical structure was suggested as 5-isoprenyl caffeic acid p-coumaric acid ester (Figure 3).

### 2.2. Effect of EEP-B on MACE Proliferation

**Figure 4:**
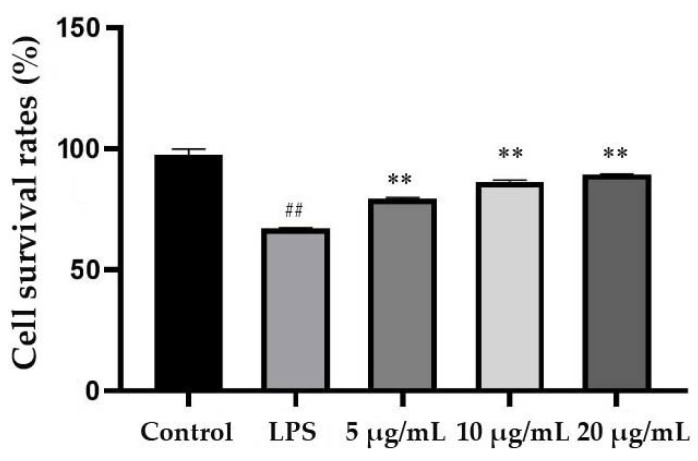
Influence of EEP-B on LPS-stimulated MACE growth. ^##^ means extremely significant difference (*p* < 0.01) between LPS-stimulated group and control and ** means extremely significant difference (*p* < 0.01) between propolis treatment group and LPS-stimulated group.

As shown in Figure 4, the survival rate of each group was measured by CCK-8 assays, and there were significant differences among all groups. The survival rate of the LPS-stimulated group was 67.35%, which was significantly lower than that of the control group. The survival rates of the cells were significantly increased when the EEP-B was at the concentrations of 5, 10 and 20 μg/mL, and the survival rate was the highest at 20 μg/mL.

### 2.3. Effect of EEP-B on the Levels of IL-6 and TNF-α

**Figure 5:**
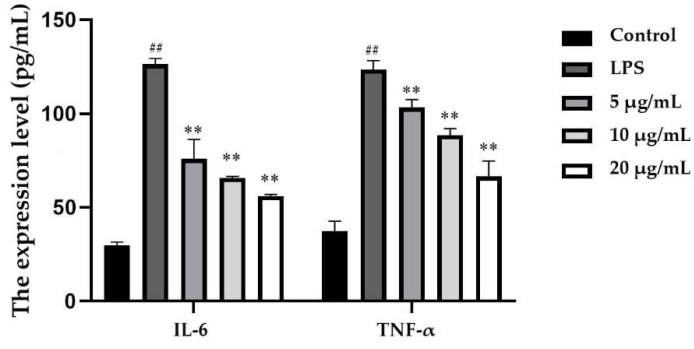
Effects of EEP-B on the levels of IL-6 and TNF-α. ^##^ means extremely significant difference (*p* < 0.01) between LPS-stimulated group and control and ** means extremely significant difference (*p* < 0.01) between propolis treatment group and LPS-stimulated group.

The expression levels of IL-6 and TNF-α were measured by ELISA. As shown in Figure 5, compared with those of the control group, the amounts of IL-6 and TNF-α secreted in the LPS-stimulated group were increased by 3.23- and 2.31-fold, respectively. After EEP-B treatment, the levels of IL-6 and TNF-α decreased significantly. At a concentration of 20 μg/mL EEP-B, the expression levels of these cytokines were the lowest.

### 2.4. Effect of Propolis on the Levels of MCP-1, ICAM-1 and VCAM-1

**Figure 6:**
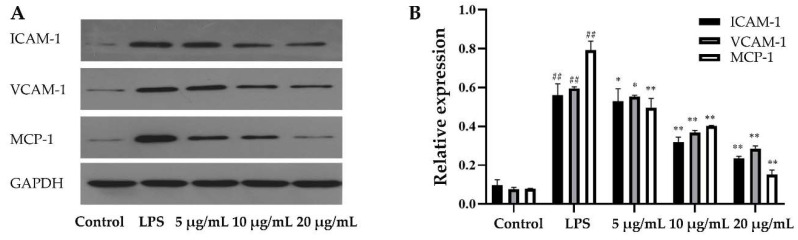
Effects of EEP-B on the levels of MCP-1, ICAM-1 and VCAM-1. (**A**). Protein expression of MCP-1, ICAM-1 and VCAM-1. (**B**). Relative expression of MCP-1, ICAM-1 and VCAM-1. ^##^ means extremely significant difference (*p* < 0.01) between LPS-stimulated group and control, * means significant difference (*p* < 0.05) and ** means extremely significant difference (*p* < 0.01) between propolis treatment group and LPS-stimulated group.

The levels of MCP-1, ICAM-1 and VCAM-1 were measured by western blotting (Figure 6). The results showed that the expression of MCP-1, ICAM-1 and VCAM-1 in the LPS group was significantly higher than that in the control group, and increased by 3.95-, 7.76- and 9.11-fold, respectively. In the EEP-B treatment group, the levels of MCP-1, ICAM-1 and VCAM-1 also decreased significantly, especially at a concentration of 20 μg/mL.

## 3. Discussion

The chemical constituents of propolis vary in different plant sources. Brazilian propolis can be classified into 13 different types according to different geographical sources, resulting in the complexity of the components [35,36]. The main chemical constituents of Brazilian green propolis are coumaric acids. In the current study, prenylated derivatives of p-coumaric acid, diterpenes and flavonoids were detected. Among these components, the level of artepillin C was much higher than that of the others, which is one of the characteristics of Brazilian propolis [37]. The level of artepillin C in propolis varies greatly depending on the geographical origin, and the samples collected from southeast Brazil are more abundant (usually 5–11%) in this component than those from other regions [37]. In addition, the components with high levels were kaempferide (7.06%), 3-isoprenyl p-coumaric acid (6.19%), pinocembrin (5.56%), diisoprenyl-p-coumaric acid isomer (4.49%), 4′-methoxy pinobanksin (4.10%) and 3-hydroxy-2,2-dimethyl-8-prenylchromane-6-propenoic (3.39%), which was consistent with previous studies [12].

Compared with poplar-type propolis, which we analysed previously, only six components, quercetin, pinobanksin, kaempferol, pinocembrin, galangin and pinobanksin-3-*O*-acetate, which are flavonoids, were the same [38]. Among them, the levels of pinobanksin, galangin and pinobanksin-3-O-acetate in poplar-type propolis were greater than 5%, 9% and 9%, respectively, while those in Brazil green propolis were all less than 0.5%, indicating great differences between these two types of propolis. The chemical composition of propolis from different regions is very complex, and new compounds have been discovered in recent years. Righi et al. identified schaftoside (apigenin-8-*C*-glucosyl-6-*C*-arabinose) in green propolis and prenylated flavonoids in Brazilian propolis [39]. Tazawa et al. identified clerodane diterpene rel-(5*S*,6*S*,8*R*,9*R*,10*S*,18*R*,19*S*)-18,19-epoxy-2-oxocleroda-3,12(*E*),14-triene-6,18,19-triol 18,19-diacetate 6-benzoate in brown propolis from Brazil [40]. In this study, the new component 5-isoprenyl caffeic acid p-coumaric acid ester was reported for the first time.

Lipopolysaccharide (LPS) is involved in the development of atherosclerosis, especially in the initial process of inflammation in atherosclerotic vessels. LPS can activate and damage endothelial cells and cause changes in the morphology and function of vascular endothelial cells [29,30]. In atherosclerotic plaque formation, vascular endothelial cell apoptosis accelerates the process of atherosclerosis [41]. In this study, LPS was used to stimulate MAECs, resulting in increased IL-6 and TNF-α expression. TNF-α and IL-6 are important inflammatory cytokines whose secretion can be stimulated by hyperglycaemia, hyperlipidaemia and other metabolic abnormalities [42,43]. IL-6 can promote the expression of adhesion molecules and other inflammatory mediators in vascular endothelial cells, and enhance the local inflammatory response, and it is an important inflammatory factor involved in tissue lesions [44]. IL-6 expression was correlated with intimal thickening and decreased elasticity [45]. Tumour necrosis factor (TNF-α) can inhibit endothelial cell proliferation, activate the nuclear factor-κB (NF-κB) signalling pathway, induce the expression of intercellular adhesion molecules such as ICAM-1 and VCAM-1, and promote monocyte infiltration to participate in the inflammatory pathological process of atherosclerosis (AS) [46,47,48].

EEP-B inhibited the expression of IL-6 and TNF-α in MAECs, which was in accordance with a previous report, suggesting that Brazilian green propolis extract has good anti-inflammatory effects by inhibiting the proinflammatory cytokines IL-6 and TNF-α [17]. Both Chinese propolis and Brazilian propolis have good immunomodulatory effects on LPS-induced inflammation. Chinese propolis modulates the suppression of autophagy and the MAPK/NF-κB signalling pathway to protect vascular endothelial cells from LPS stimulation [49]. Brazilian green propolis exerts strong anti-inflammatory effects by inhibiting the production of many cytokines, such as IL-1α, IL-1*β*, IL-4, IL-6, TNF- α and MCP-1, in stimulated J774A cells [39]. Similar to green propolis, red propolis extract can also inhibit the release of inflammatory cytokines, including TNF-α, IL-1*β*, CXCL1/KC and CXCL2/MIP-2. In addition, Brazilian propolis can prevent neutrophil migration into the peritoneal cavity, and reduce leukocyte rolling and adhesion on the mesenteric microcirculation [50].

Propolis plays an important role in the prevention and treatment of cardiovascular diseases [20,51,52]. In initial atherosclerotic lesions, propolis polyphenol extracts improved the lipid profile and decreased the atherosclerotic lesion area [53], as well as reducing the total cholesterol (TC), low density lipoprotein-cholesterol (LDL-C), triglycerides and thiobarbituric acid-reactive substance concentrations [54]. The propolis component chrysin has been confirmed to inhibit plasma plasminogen activator inhibitor 1 (PAI-1) production, and could be used to treat or prevent thrombotic disorders [25]. The expressions of ICAM-1, VCAM-1 and MCP-1 were rapidly upregulated upon stimulation with the inflammatory factor LPS. The high expression of ICAM-1 enhances the adhesion of monocytes to endothelial cells, VCAM-1 can promote monocyte adhesion to endothelial cells, and monocyte chemotactic protein-1 (MCP-l) can activate monocytes by binding with receptors [55,56]. The current study revealed that Brazilian green propolis could reduce the expression levels of ICAM-1, VCAM-1 and MCP-1, in order to inhibit the migration of monocytes from the blood circulation to the intima of the blood vessels, reduce the aggregation of foam cells and lipids in the arterial wall, and inhibit the chemotaxis of monocytes to the lesion site, thereby reducing the accumulation of lipids in the intima and damage to the intima of blood vessels.

## 4. Materials and Methods

### 4.1. Preparation of Experimental Samples

Brazilian green propolis was supplied by Fujian Shenfeng Technology Development Co., Ltd. (Fuzhou, China). Propolis was extracted using ethanol (70%, *v*/*v*) according to a previously described method [38]. The ultrasonic wave method was used to extract propolis, and beeswax was removed. The ethanol extracts of Brazilian propolis were named EEP-B.

### 4.2. Chemical Analysis of EEP-B

The quantification of total polyphenol and phenolic contents and chemical composition analysis were also performed according to a previously described method [28]. The quantification of total polyphenols was performed using the Folin–Ciocalteu method, and gallic acid was used as a standard. The total flavonoid content was determined as described in the national standards for propolis in China (GB/T 24283-2018), and rutin was used as a standard. UPLC-ESI-MS analysis was performed on a Waters UPLC system, and the mobile phases consisted of 0.1% formic acid in water (A) and acetonitrile (B). The mass spectra were obtained using a Waters definition accurate-mass quadrupole time-of-flight (Q-TOF) Xevo G2-XS mass spectrometer (Waters Ltd., Elstree, Hertfordshire, UK), which was equipped with an ESI source, in both positive and negative ion modes.

### 4.3. Cell Culture

MAECs and reagents for cell culture were purchased from Procell Life Science and Technology Co. (Wuhan, China). Cells were cultured to a density of 1 × 10^5^ /mL at 37 °C in 5% CO_2_ and then divided into the following five groups: cells not treated with LPS or propolis that were used as control, cells stimulated with LPS (10 μg/mL), and cells stimulated with LPS (10 μg/mL) that were treated with different concentrations of EEP-B (5, 10 and 20 μg/mL).

### 4.4. Cell Viability Assay

Cells were added to each well at a density of 1 × 10^5^ /mL and cultured for 24 h, then 10 μL of Cell Counting Kit-8 (CCK-8) solution (Beyotime Biotechnology, Shanghai, China) was added and further cultured for 4 h at 37 °C in 5% CO_2_. Then, the absorbance was measured at 450 nm. The cell viability (%) was calculated by the following formula: (absorbance of experimental wells/absorbance of control wells) × 100%.

### 4.5. ELISA Analysis of IL-6 and TNF-α Expression

The cells were cultured for 24 h and used for enzyme-linked immunosorbent assay (ELISA) analysis. The supernatant of each group was analysed according to the procedures of the ELISA kit (Neobioscience, Shenzhen, China). The absorbance of each sample was measured at 450 nm by a microplate reader (Diatek, Wuxi, China), and the concentrations of IL-6 and TNF-α were calculated.

### 4.6. Western Blot Analysis of ICAM-l, VCAM-1 and MCP-1 Expression

The cells were cultured for 24 h and used for western blot analysis. Radio immunoprecipitation assay (RIPA) buffer was used to lyse the cells, and the protein concentration was measured by the bicinchoninic acid (BCA) method using the Pierce^TM^ Rapid Gold BCA Protein Assay Kit (Elabscience, Wuhan, China). Total protein samples from each group were extracted for sodium dodecyl sulphate polyacrylamide gel electrophoresis (SDS-PAGE). The proteins were transferred to a polyvinylidene fluoride (PVDF) membrane, incubated with the primary antibody against the target protein overnight at 4 °C and washed 5 times using Tris-buffered saline Tween-20 (TBST). Then, the secondary antibody was added and incubated for 1 h at room temperature, and the membrane was washed with TBST again. Finally, Clarity Western ECL Substrate was used for luminescence detection, and AlphaEaseFC was used to analyse the gel image. Glyceraldehyde-3-phosphate dehydrogenase (GAPDH) was used as a loading control.

### 4.7. Statistical Analysis

All experiments were performed in triplicate. The data are expressed as the mean ± standard deviation (*n* = 3). Statistical analysis was performed using t-tests and one-way ANOVA with SPSS software. Differences of *p* < 0.05 were considered statistically significant.

## 5. Conclusions

Propolis possesses rich chemical components and robust anti-inflammatory activity. In this study, the chemical composition of Brazilian green propolis was analysed by UPLC-Q-TOF-MS. A total of 28 compounds was identified, and a new component, 5-isoprenyl caffeic acid p-coumaric acid ester, was tentatively identified for the first time. In addition, EEP-B showed strong anti-inflammatory effects by inhibiting the levels of the cytokines TNF-α and IL-6, and protecting MAECs by regulating ICAM-1, VCAM-1 and MCP-1 expression. This study provides new insights into the chemical components and the anti-inflammatory activities of Brazilian green propolis.

## Figures and Tables

**Table 1:** Main components of the ethanol extracts of Brazilian propolis (EEP-B).

No	tR (min)	λmax (nm)	Selected Ion	Formula	Measured Mass	Calculated Mass	Mass Error	MS/MS Fragmentation	Compound Name	Relative Area (%)
1	4.62	237,310	[M−H]	C_9_H_8_O_3_	163.0400	163.0395	0.5	163.0400, 119.0502, 93.0368	*p*-Coumaric acid ^a,c^	1.31%
2	6.18	324	[M−H]	C_25_H_24_O_12_	515.1191	515.1190	0.1	353.0868, 91.0554, 179.0342, 135.0444	Dicaffeoylquinic acid isomer ^a,d^	0.48%
3	6.64	324	[M−H]	C_25_H_24_O_12_	515.1193	515.1190	0.3	353.0871, 191.0555, 179.0343, 135.0445	Dicaffeoylquinic acid ^a,d^	1.31%
4	9.06	253,372	[M−H]	C_15_H_10_O_7_	301.0365	301.0348	1.7	243.02886, 151.00304	Quercetin ^a,c^	0.29%
5	10.96	292	[M−H]	C_15_H_12_O_5_	271.0613	271.0606	0.7	271.0613, 253.0504	Pinobanksin ^a,c^	0.19%
6	11.26	292	[M−H]	C_16_H_14_O_6_	301.0714	301.0712		301.0714, 283.0603, 268.0367, 152.0107	4′-methoxy Pinobanksin ^a,e^	4.10%
7	11.40	265,365	[M−H]	C_15_H_10_O_6_	285.0400	285.0399	0.1	285.0400, 227.03402	Kaempferol ^a,b^	0.30%
8	11.58	251,365	[M−H]	C_16_H_11_O_7_	315.0505	315.0505	0	315.0505, 300.0266, 243.0292	Quercetin-methyl ether ^a^	0.43%
9	13.80	326	[M−H]	C_14_H_16_O_4_	247.0971	247.0970	0.1	247.0971, 203.10683	5-isoprenyl caffeic acid ^a,f^	0.53%
10	15.52	315	[M−H]	C_14_H_16_O_3_	231.1025	231.1021	0.4	187.1121, 132.0575	3-isoprenyl-*p*-Coumaric acid ^a,e^	6.19%
11	17.11	313	[M−H]	C_19_H_24_O_4_	315.1597	315.1596	0.1	315.1597, 271.1692, 253.1591, 198.1043	3-hydroxy-2,2-dimethy-8-prenylchromane-6-propenoic ^a,e^	3.39%
12	17.47	324	[M−H]	C_14_H_16_O_4_	247.0971	247.0970	0.1	247.0971, 179.0341, 161.0236, 135.0443	Caffeic acid isoprenyl ester ^a,c^	0.46%
13	19.00	286	[M−H]	C_15_H_12_O_4_	255.0662	255.0657	0.5	213.0552, 151.0031, 107.0134	Pinocembrin ^a,c^	5.56%
14	19.24	318	[M−H]	C_19_H_24_O_4_	315.1597	315.1596	0.1	271.1675, 253.1589, 198.1041	3-hydroxy-2,2-dimethy-8-preylchromane-6-propenoic isomer ^a,e^	0.45%
15	19.65	265,361	[M−H]	C_15_H_10_O_5_	269.0456	269.0450	0.6	211.03914, 145.0288, 117.0340	Galangin ^a,b^	0.53%
16	20.35	265,365	[M−H]	C_16_H_11_O_6_	299.0557	299.0556	0.1	284.0320, 151.0029, 107.0132	Kaempferide ^a,c^	7.06%
17	20.68	292	[M−H]	C_17_H_14_O_6_	313.0745	313.0712	3.3	253.0505, 119.0498	Pinobanksinr-3-*O*-acetate ^a,c^	0.37%
18	21.18	269, 363	[M−H]	C_17_H_14_O_7_	329.0665	329.0661	0.4	314.0424, 299.0180, 271.02431	Quercetin-dimethyl ether ^a,c^	2.52%
19	24.25	319	[M−H]	C_23_H_22_O_6_	393.1343	393.1338	0.5	349.1436, 247.0967, 163.0392, 145.0289, 117.0340	5-isoprenyl caffeic acid-*p*-coumaric acid ester ^a^	0.62%
20	25.84	318	[M−H]	C_19_H_24_O_4_	315.1600	315.1596	0.5	245.1170, 201.1279, 146.0731	3-hydroxy-2,2-dimethy-8-preylchromane-6-propenoic isomer ^a,e^	0.43%
21	27.17	287	[M−H]	C_19_H_24_O_3_	299.1647	299.1647	0	255.1748, 187.1117, 161.0601	Diisoprenyl -*p*-Coumaric acid isomer ^a,e^	4.49%
22	32.88	314	[M−H]	C_19_H_24_O_3_	299.1652	299.1647	0.5	255.1750, 201.1238, 145.0652	Artepillin C (3,5-diisopentenyl-4-hydroxycinnamic acid) ^a,e^	35.68%
23	38.36	279	[M−H]	C_23_H_24_O_4_	363.1588	363.1596	−0.8	231.1021, 187.1122, 149.0621	3-Prenyl-4-(dihydrocinnamoyloxy)-cinnamic acid ^a,d^	2.44%
24	38.51	308	[M−H]	C_28_H_32_O_5_	447.2175	447.2171	0.4	297.1492, 253.1592, 198.1041, 149.0603, 105.0704	(E)-3-[2,3-dihydro-2-(1-methylethenyl)-7-prenyl-5-benzofuranyl]-2-propenoic acid ^a,d^	2.27%
25	40.73	-	[M−H]	C_30_H_48_O_4_	471.3485	471.3474	1.1	517.3542, 471.3485, 407.3318	triterpenes	0.94%
26	42.48	-	[M−H]	C_30_H_46_O_4_	469.3323	469.3318	0.5	515.3377, 469.3323, 423.3260	triterpenes	0.17%
27	43.25	-	[M−H]	C_30_H_48_O_4_	471.3480	471.3474	0.6	517.3436, 471.3481, 339.1994	triterpenes	0.24%
28	43.68		[M−H]	C_30_H_46_O_4_	469.3322	469.3318	0.4	515.3377, 469.3322, 407.3314	triterpenes	2.26%

tR(min): retention time (min); λmax: the maximum absorption wavelength. ^a^ confirmed with MS fragmentation; ^b^ Confirmed with the standard; ^c^ confirmed with references [31]; ^d^ confirmed with references [32]; ^e^ confirmed with references [33]; ^f^ confirmed with references [34].
